# Prospects for atomic resolution in-line holography for a 3D determination of atomic structures from single projections

**DOI:** 10.1186/s40679-017-0041-6

**Published:** 2017-02-06

**Authors:** F. -R. Chen, C. Kisielowski, D. Van Dyck

**Affiliations:** 1 Department of Engineering and System Science, National Tsing-Hua University, Hsin Chu, Taiwan; 2 The Molecular Foundry and Joint Center for Artificial Photosynthesis, Lawrence Berkeley National Laboratory, One Cyclotron Rd., Berkeley, CA 94720 USA; 3 Department of Physics, EMAT, University of Antwerp, 2020 Antwerp, Belgium

**Keywords:** In-line holography, Exit wave function, Atomic resolution tomography, 3D molecular imaging

## Abstract

It is now established that the 3D structure of homogeneous nanocrystals can be recovered from in-line hologram of single projections. The method builds on a quantitative contrast interpretation of electron exit wave functions. Since simulated exit wave functions of single and bilayers of graphene reveal the atomic structure of carbon-based materials with sufficient resolution, we explore theoretically how the approach can be expanded beyond periodic carbon-based materials to include non-periodic molecular structures. We show here theoretically that the 3D atomic structure of randomly oriented oleic acid molecules can be recovered from a single projection.

## Background

The ultimate goal of electron microscopy is to act as a communication channel between structure and properties of materials. Certainly, all material properties are determined by the atom arrangement in three dimensions (3D), which are especially rich if complex atom configurations are considered that are intrinsic to composites such as combinations of catalysts and molecules. There has been significant progress towards electron tomography of crystalline and radiation hard matter using aberration-corrected scanning transmission electron microscope (STEM) [1, 2] and transmission electron microscope (TEM) [3, 4]. Contributing to these examples, our TEM approach [3, 4] allows for quantitative 3D structure determination with atomic resolution from only one viewing direction if it is chosen close to zone axes orientations of the crystalline matter. This goal is achieved by reconstructing electron exit wave functions from image focal series [5, 6] that capture the dynamic nature of electron scattering and the pronounced dependence of local image contrast on focus. In case of crystalline materials, our interpretation bases on the Channeling Theory that provides the number of atom in a column and on the fact that the *z*-coordinate of atoms at the exit surface can be determined locally from intensity maxima of propagated wave functions to a precision that exceeds interatomic distances. As a starting point for a 3D characterization of carbon-based materials, we summarize essential features of the tool by modeling a single and bi-layer of graphene at atomic resolution [3], which is a material of outstanding radiation hardness unlike the majority of single molecules [7, 8]. Beyond investigations of radiation hard periodic matter, the approach offers intrinsic advantages to study beam-sensitive materials such as catalysts and molecules because dose-rate dependences can be exploited to help reducing beam-sample interactions so that atomic resolution and single atom sensitivity may be achieved without altering the pristine structure of radiation sensitive matter [8]. In this paper, we demonstrate the 3D information of the atomic position in encoded in the exit wave function reconstructed from simulated focus series images of single/double layer graphene without considering the influence of the noise. The noise will affect the precision of the focus and mass determination which has been demonstrated in our earlier publication [4] with experimental exit wave functions. We further explore the possibility to recover the non-periodic, 3D structure of molecules from simulated focus series of the oleic acid molecules.

## Methods

For the crystalline case, our method exploits the Channeling Theory [9] that allows treating an image as an array of individual atom columns up to thicknesses of tens of nm and neglects the interaction between columns. In this model, the atoms of a column act as weak lenses that focus an electron wave periodically with increasing depth. According to the Channeling Theory [9, 10], the exit wave function Ψ_e_(**r**,t) at a particular image plane with small distance Δf from the exact exit surface, where the scattered waves from neighboring columns are assumed to not interfere, can be expressed analytically as

1 $$ \varPsi_{\text{e}} \left( {{\mathbf{r}}, \, t} \right) = \varPsi \left( {{\mathbf{r}},0} \right) + \varPhi_{{ 1 {\text{s}}}} \left( {\mathbf{r}} \right)\left( {{\text{e}}^{{ - i{\text{Et}}}} - 1} \right)\left( { 1- {\text{e}}^{{ - i\alpha \Delta {\text{f}}}} } \right), $$

where *t* is the mass thickness of a column; Ψ(**r**,0) is the incident wave, and Φ_1s_(**r**) is the 1 s eigenstate of the projected electrostatic potential of the atom column with eigen-energy E. α is a constant. The mass thickness is given by the number of atoms and their equidistant spacing in a column and E is proportional to their atomic number Z. It measures the phase change per atom in units of rad/atom. Therefore, Et is the total phase change of an entire atom column.

**Fig. 1:**
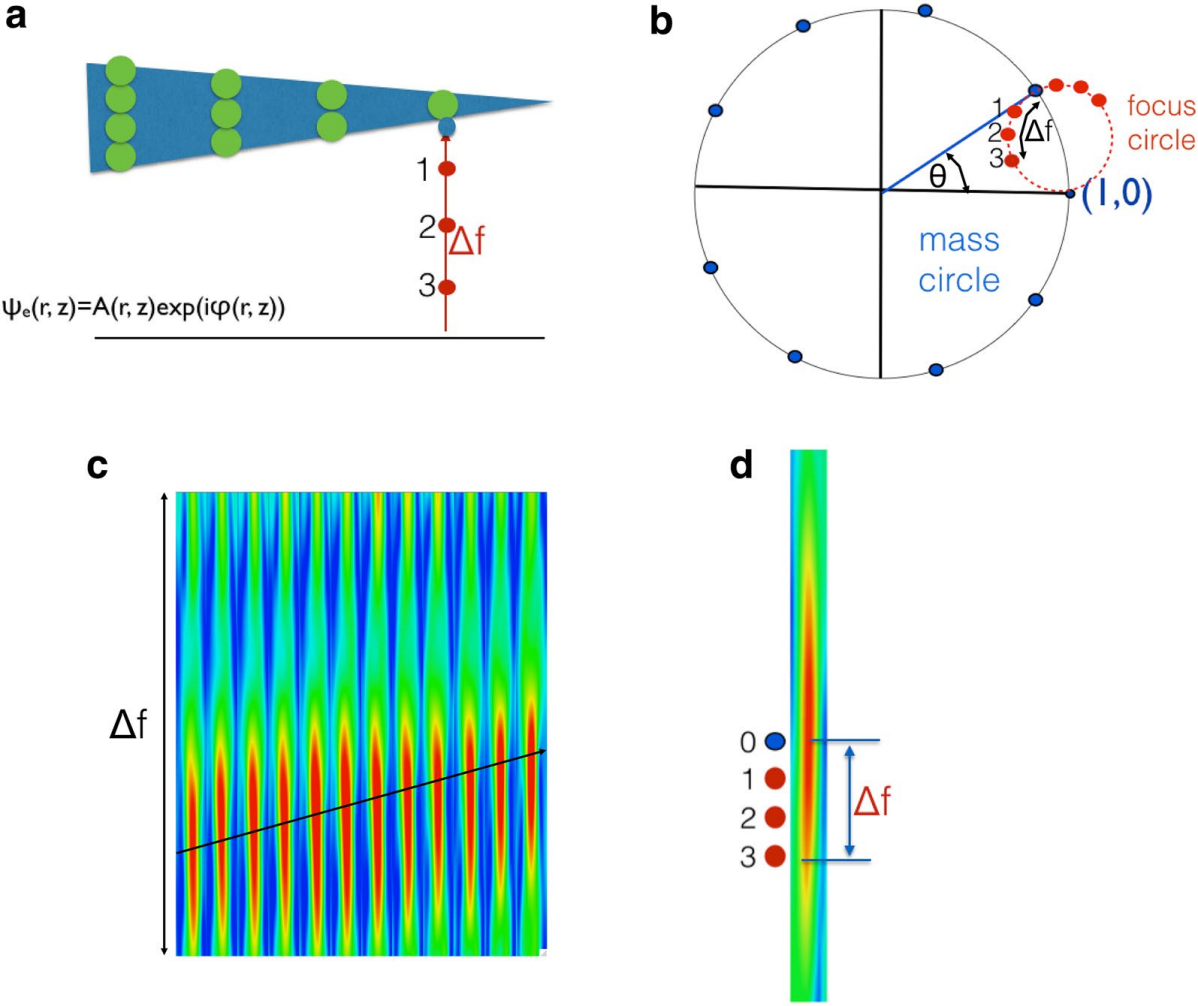
**a** Model of a wedge-shaped crystal whose* columns* contain a different number of atom. The exit surface of each column differs in *z*-height (beam direction) with respect to a common image plane. **b** Representation (Argand plot) of the complex pixel values at the centers of a* column*. *Blue dots* move along the *black circle* as the number of atoms in a column increases; *red dots* move along the * red, dash line* as the propagation distance Δf changes from 1 to 3. **c** The propagation intensity for a set of experimental gold columns [3]. **d** The “true” focus value (*blue*) is detected if the propagation intensity reaches a maximum

**Fig. 2:**
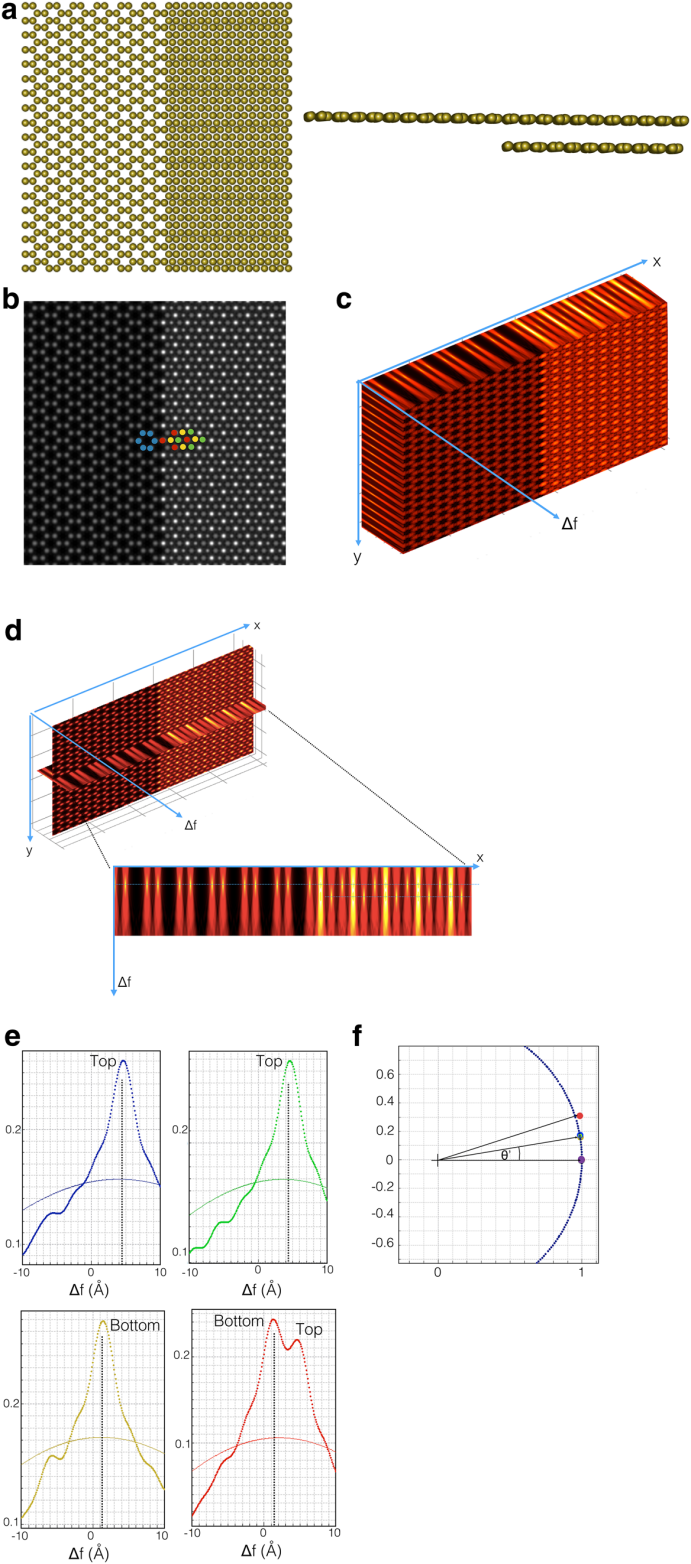
A structural model of a graphene single/double layer. **a** The *left figure* is a plan view and the *right figure* shows a cross section of the structural model. **b** Phase of the simulated exit wave function. There are four geometrically different atom configurations: C-atoms in the *top* layer (*blue dots*), C-atoms in the *upper* layer that are not superimposed on those in the *bottom* layer (*green dots*), C-atoms in the *bottom* layer that are not superimposed on the *top* layer (*yellow dots*) and two superimposed C-atoms (brightest peaks, *red dots*) **c** The 3D propagation intensity. **d** An intensity slice across a *x*–*z* plane including the four geometrical configurations. **e** The intensity profile along *z*-direction for four different types of atom for two different g_*max*_ = 4Å^−1^ (*dot line*) and 1Å^−1^ (*solid line*), respectively. The hidden atom can be clearly revealed in the intensity profile with g_*max*_ = 4Å^−1^. It smears out in the intensity profile with g_*max*_ = 1Å^−1^. **f** Mass circle of the four different atom configurations

As shown in Fig. 1a, Δf is the local distance between the exit surface of the column and the image plane of the holographically reconstructed wave function. Every pixel in the exit wave function is a complex number and the Channeling Theory provides an intuitive way to interpret the underlying physics, which can be visualized graphically by plotting the complex pixel values in the Argand plot of Fig. 1b. It is clear that the two factors (e^−*i*Et^ − 1) and (1 − e^−*i*αΔf^) from Eq. (1) describe two circles which yield two independent (approximately orthogonal) coordinates: the black circle yields the total mass of the column (Et), and the red-dashed circle yields the defocus (αΔf)) between an atom at the exit surface of the sample and the image plane of the reconstruction (Fig. 1b). The exit wave function at the exact exit surface of the sample is represented by the blue dots of the mass circle in Fig. 1b and the total number of atoms contained in a column is determined by the phase angle θ’. It is defined with respect to the vacuum wave at (1,0) and is proportional to Et. The geometry of samples, however, usually does not include exit surfaces that are atomically flat. For example, it is wedge shaped in Fig. 1a. Hence, it is impossible to focus the exit wave functions of arbitrarily shaped samples into a single image plane without introducing local focus changes Δf = Δf(*x*,*y*). We compensate for locally varying focus values by propagating the image of each atomic column into a common image plane along the red-dashed defocus circle in Fig. 1a. For convenience, discrete red dots 1–3 are indicated in Fig. 1 that mark different propagation distances. If one tracks the intensities for each propagating step, they form an ellipsoid (Fig. 1c) with a maximum at the focused, “true” *z*-position where the total mass circle and the defocus circle intersect as shown in Fig. 1c and d. Therefore, the local propagation distances Δf(*x*,*y*) measure the geometrical shape of the bottom sample surface with respect to the common image plane. It is noted that interference between neighboring columns can occur for large propagation distances and that they can be affected by residual lens aberrations that we minimize during the reconstruction process using numerical phase plates. Cross sections of experimentally determined propagation ellipsoids from adjacent columns of a gold nano-bridge are shown in Fig. 1c [4], and Fig. 1d illustrates when the wave reaches the exact exit surface (the blue dots) that coincides with the intensity maximum. From Argand plots of each column, we can deduce the (*x*, *y*) position of the column, its z-coordinate with sub-Angstrom precision that is given by Δf(*x*,*y*), the total mass of the column, and the residual lens aberrations [3, 11]. By combining this information, we can then reconstruct the 3D shape of samples from one projection with single atom sensitivity [4]. The method is self-consistent and includes the following steps:

- Determination of the true “*z*” height (focus) of the exit surface of a column from the common image plane using the maximum propagation intensity (MPI) as a criterion as shown in Fig. 1b. This is equivalent to determine the distance Δf between the a red dot and the blue dot in the same defocus circle.
- Refining the “*z*” height (Δf) using the Big-Bang scheme [3].
- Correcting the focus of each column by wave propagation to create an image where the red and the blue dot coincide in Fig. 1b and create the mass circle.
- Since we evaluate peak-to-valley phase values, the focus corrected wave Ψ is calibrated with another wave φ determined in the “valley” surrounding the atom columns. The valley value corresponds to “zero mass” and it is expected to be close to vacuum wave (1,0).
  $$ \varPsi \left( {\text{norm}} \right) = \psi /\varphi $$
  Ψ(norm) is the normalized focus corrected wave function so as to corrected by the mean inner potential of the crystal.
- The column mass is given by the phase angle θ’ between the normalized focus corrected wave functions and the vacuum wave which is close to (1,0). See Fig. 2f. The θ’ is proportional to the Et.

In the next section, we first illustrate for the case of single and bi-layer graphene that our approach allows resolving interatomic distances in beam direction [3, 4], which is a capability that allows extending the application to reveal the 3D structure of molecular networks if they are captured in low dose-rate image series that can maintain their pristine structure [13].

## Results and discussion

**Fig. 3:**
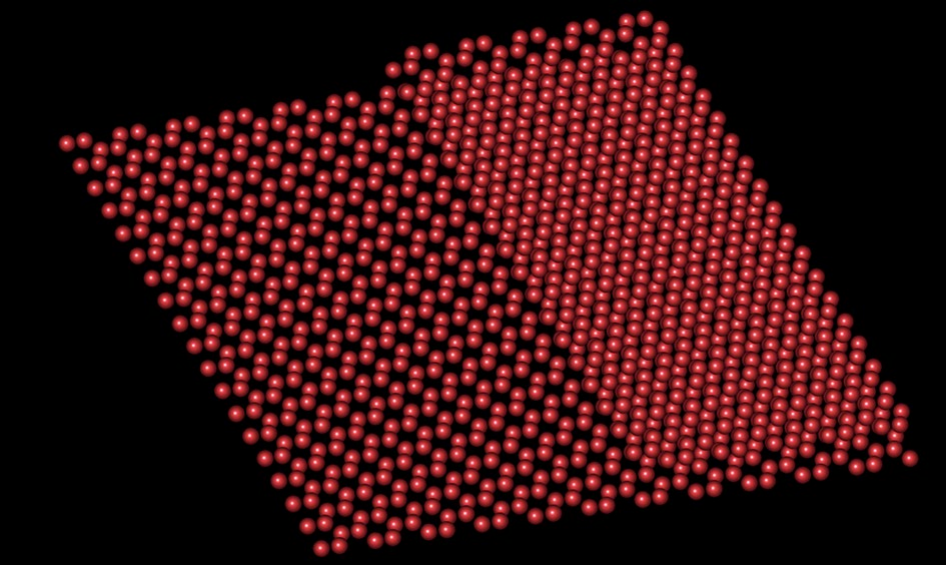
3D reconstruction of the single-/double-layer graphene using the information from Fig. 2

A structural model of single/double layer graphene is shown in Fig. 2a. The exit wave function (modulus and phase) is simulated for 80 keV with a largest diffraction vector g_*max*_ = 1.5Å^−1^ and its phase is shown in Fig. 2b. Within the field of view, there are four atom configurations of different geometries: atoms that reside in the upper layer (blue dots), atoms in the upper layer that do not superimpose on atoms of the bottom layer (green dots), atoms in the bottom layer that do not superimpose on atoms the of top layer (yellow dots), and those that superimpose on both layers (brightest peaks, red dots). For a particular focus setting, the intensity of the exit wave function is displayed in Fig. 2c together with the various propagation intensities in beam direction. In Fig. 2d, one particular intensity slice across the *x*–*z* plane is displayed that includes all four atom configurations. They are clearly revealed by locating the intensity maxima, and the separation of the two different layers is readily observable. Although the resolution of exit wave function with g_*max*_ = 4Å^−1^ may not be achievable with the present instrument, to demonstrate the atomic position in 3D is indeed encoded in the exit wave function, intensity profiles of the four atom configurations along the *z*-direction (the focus direction) are displayed in Fig. 2e for two different values g_*max*_ = 4Å^−1^ (dot line) and 1Å^−1^ (solid line) that describe resolution. Surely, the width of the intensity distributions increases dramatically as a result of resolution loss. Nevertheless, intensity maxima consistently peak at the atom position even though the signal of superimposed atoms vanishes for the poorer resolution g_*max*_ = 1Å^−1^. Figure 2f shows the Argand plot of the mass circle. Exploiting these measurements, we have created the tomogram that is shown in Fig. 3. Experimentally, one expects that the radius of the mass circle will be modified by thermal vibrations (Debye–Waller factors) and by electron beam-induced sample excitations [4, 11].

**Fig. 4:**
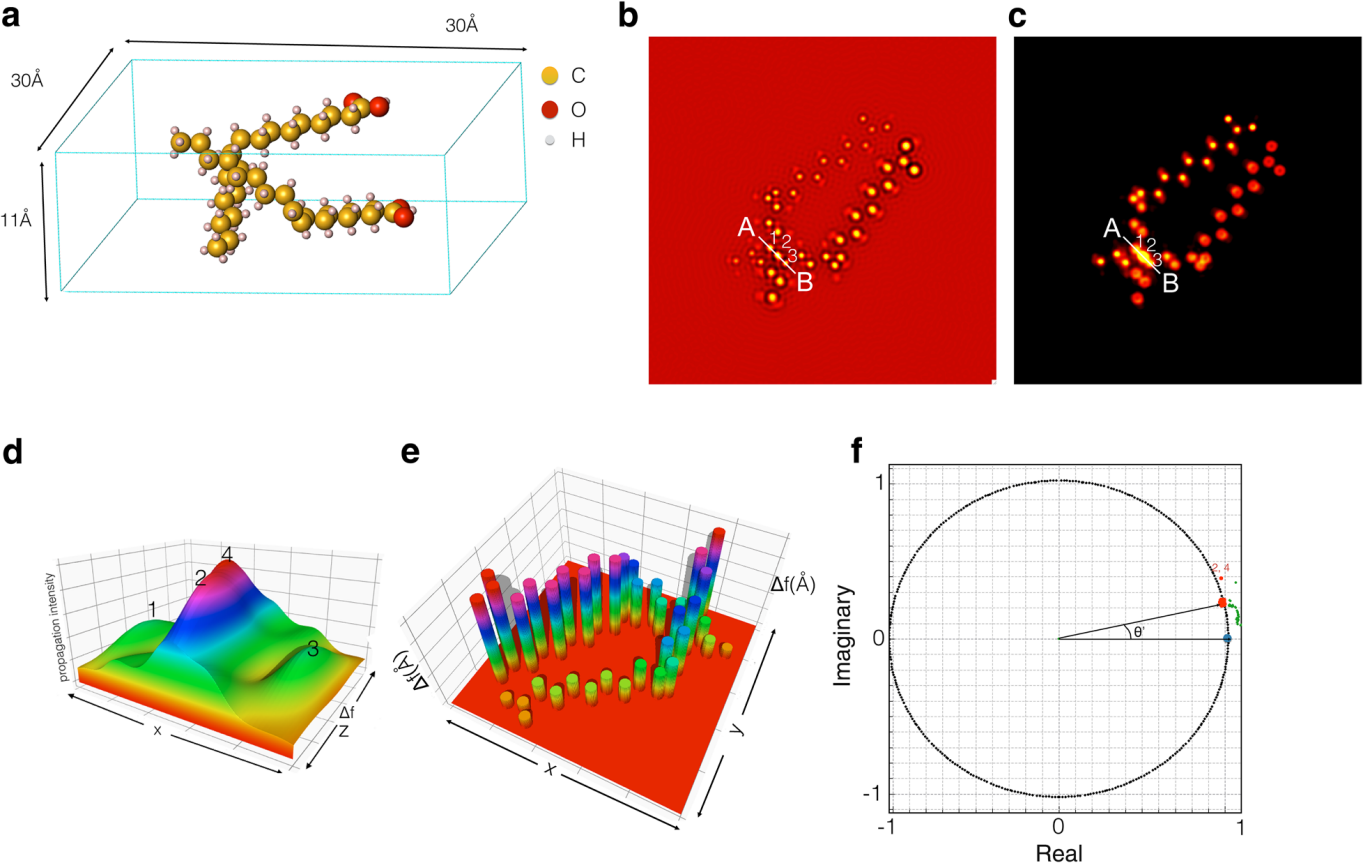
**a** Structural model of the two crossing oleic acid molecules. **b**, **c** The real and imaginary parts of the simulated electron exit wave function. **d** Propagation intensity along the A–B segment. **e** Focus map. **f** Mass circle (Argand plot). In this plot, the *green dots* are the original waves and the *red points* are already corrected for defocus so that the points are concentrated on the* mass circle*

**Fig. 5:**
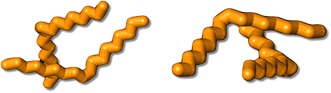
3D reconstruction of the oleic acid molecules

Next, we test the possibility to extend the method and include non-periodic/radiation sensitive objects. For this purpose, we focus on oleic acid molecule that were recently used to control the averaged spacing among FePt nanoparticles [12, 13]. Their chemical structure is given by CH_3_(CH_2_)_7_CH=CH(CH_2_)_7_COOH. Here, we consider the V-shaped *cis*-form of the molecule [14]. Figure 4a shows the two oleic acid molecules that cross each other. For a potential comparison with experiments [12], the exit wave is simulated with g_*max*_ = 1.8Å^−1^, which is close to the resolution limit of the TEAM 0.5 microscope operated at 80 kV [7]. Figures 4b and c depict the real and imaginary parts of the simulated exit wave function of the structural model shown in Fig. 4a. The white lines in Fig. 4b and c cross three atom or column positions marked as #1, #2, and #3 and Fig. 4d shows the propagation intensity from these columns. The graph reveals the extra atom hidden in the location #2, and their separation in z-direction is determined to be 3.9 Å in agreement with the input structure. The *z*-height of all atoms or columns is shown in the focus map of Fig. 4e. It is seen at the two molecules which are tilted in opposite direction across each other. As expected, the tilt is recognized in an Argand plot (Fig. 4f) by the elongated distribution of the original complex data points (the green dots) that reveal the location of atoms in a different height. After a focus correction, the green dots move onto the mass circle that explicitly provides the number of atoms and their chemical identity. The red dot marked as (2,4) corresponds to the position #2 that contains two atoms. Combining the information given by the focal map (*z*-height) and the mass circle (number of atom), a three-dimensional model of two molecule chains can be reconstructed (Fig. 5) that faithfully reproduces the input structure.

## Conclusions

This contribution refines the methodology to reconstruct 3D shape of nanomaterials at atomic resolution from a single projection of electron exit wave functions. Further, it is extended to demonstrate that it is possible to reconstruct non-periodic, molecular structures in three dimensions that can be recorded in low dose-rate conditions. Therefore, the approach is exceptionally well suited to image single molecules and reconstruct soft/hard matter components in 3D.
